# Forgiveness: A Key Component of Healing From Moral Injury?

**DOI:** 10.3389/fpsyt.2022.906945

**Published:** 2022-07-13

**Authors:** Suzette Brémault-Phillips, Terry Cherwick, Lorraine Alison Smith-MacDonald, John Huh, Eric Vermetten

**Affiliations:** ^1^Faculty of Rehabilitation Medicine, Department of Occupational Therapy, University of Alberta, Edmonton, AB, Canada; ^2^HiMARC, Faculty of Rehabilitation Medicine, University of Alberta, Edmonton, AB, Canada; ^3^Royal Canadian Chaplain Service, Department of National Defence, Edmonton, AB, Canada; ^4^Department of Psychiatry, Leiden University Medical Center, Leiden, Netherlands

**Keywords:** Moral Injury, forgiveness, relationships, intervention, PTSD—posttraumatic stress disorder, healing

## Abstract

Service members and veterans can be exposed to potentially traumatic and morally injurious experiences (PMIEs) including participating in, witnessing, or failing to prevent an act(s) that transgresses their core beliefs. Violation of one's deeply held morals and values can be profoundly distressing and shatter one's sense of self at the deepest level. Relationships with self, others, the world, and for some, the Sacred, can also be fractured. Post-Traumatic Stress Disorder (PTSD) and/or Moral Injury (MI) can result. Left unresolved, MI can leave individuals struggling with guilt, shame, cognitive dissonance, and negative self-attributions. A holistic approach that addresses the psychological and spiritual harm associated with MI is warranted. We wonder if forgiveness can help individuals struggling with MI to address the harm caused by actions or inactions, release negative emotions, and mend relationships. Commonly used by Spiritual/Religious (S/R) Leaders, forgiveness practices are increasingly being explored by Mental Health Professionals as a complement to evidence-based treatment approaches. This article provides case examples that illustrate the use of forgiveness practices that promote recovery and identifies programs used in clinical practice that incorporate forgiveness. Research is yet needed to better understand the importance of forgiveness in the treatment and healing of PTSD and/or MI. This requires an interdisciplinary discourse between Mental Health Professionals and S/R Leaders working in the field of MI. Such engagement and integrated use of forgiveness practices may yield improved outcomes not only for service members and veterans, but for all those struggling as a result of PTSD and/or MI.

## Introduction

Deep moral woundedness, more recently termed “Moral Injury” (MI), can be a key aspect of post-traumatic stress injuries (1, 2). Moral Injury has been defined as a “particular trauma syndrome including psychological, existential, behavioral, and interpersonal issues that emerge following perceived violations of deep moral beliefs by oneself or trusted individuals” (3). MI is preceded by exposure to potentially morally injurious experiences (PMIEs) such as participating in, witnessing, or failing to prevent an act that transgresses one's beliefs and values (4). Feelings of guilt, shame, and betrayal; emotional dysregulation and negative self-attributions are associated with MI, as are a shattering of one's sense of self, meaning, and purpose; corrosion of one's soul; and erosion of one's sense of values, beliefs, and a benevolent orderly world (1–18). Relationships can also be fractured with self, others, and for some, the Sacred—where the Sacred is understood as what is most meaningful and significant to a person, which would include concepts of the transcendent, holy, divine, ultimate being, and mystery (19–24).

We wonder whether individuals experiencing MI may benefit from an integrated psychological and spiritual approach, and propose consideration of forgiveness as a means of facilitating recovery from MI. During psychotherapy, Mental Health Professionals may see clients struggling with MI-related unforgiveness but without skills to address it (25). Spiritual/Religious (S/R) Leaders on interdisciplinary teams may support healing given their attunement to the S/R needs of service members and veterans and familiarity with forgiveness. S/R Leaders can establish trusting, non-judgmental relationships; convey that no topic is off-limits for thoughtful and compassionate discussion; and support reintegration into a moral community (be it religious, secular, familial, or other) (26–28).

The purpose of this article is to examine ways in which giving and receiving forgiveness may help restore one's sense of self and relationships. Additionally, it explores forgiveness in the context of MI from an interdisciplinary perspective that integrates mental and S/R domains and forgiveness practices as interventions.

## Forgiveness Practices as Interventions

Forgiveness is a complex neurocognitive, affective, and spiritual process (29). A literature search yielded growing attention to forgiveness as a process and practice and increasing interest in it across disciplines (30). While no universally-accepted definition of forgiveness has been found, numerous understandings of forgiveness have emerged and critical ingredients of the process have been identified. Hartz isolated letting go of anger and reducing negative thoughts and feelings about self and others as being central to forgiveness (31), with forgiveness fundamentally calling for a shift in motivation away from retaliation and avoidance (unforgiveness) and toward undeserved goodwill for the perceived wrongdoer (forgiveness). Importantly, forgiveness occurs along a continuum from no forgiveness through complete forgiveness and potential relational restoration (31). This process can positively impact personal wellbeing and play a vital role in restoring social relationships (32). Seeking and receiving forgiveness has helped people find wholeness, offer unconditional forgiveness to others and themselves, and move them forward in their recovery (33–36).

Various approaches to forgiveness may be effective when dealing with MI. One comprehensive understanding of forgiveness is Enright's triadic forgiveness approach (37) which encompasses (i) forgiveness of the self, (ii) giving and receiving forgiveness from others, and (iii) forgiveness of the Sacred (38). Understanding the forgiveness triad and associated practices and processes can help Mental Health Professionals and S/R Leaders support recovery from shame and guilt (39, 40) that can compromise a person's relationships horizontally (with self and others) and vertically (with the Sacred) (20, 33, 41, 42).

Giving and receiving horizontal and vertical forgiveness is reflected in Canadian General (retired) Dallaire's PTSD journey following the 1994 Rwandan Massacre (43). Dallaire recognized that MI-related guilt and anger impeded his healing. His relationships with self and others were fractured and his faith destroyed. He noted: “*(faith) was something that I... fought against in the post-Rwanda genocide period. God had abandoned 800,000 Rwandans, my force, myself, and did absolutely nothing to stop it”* (44). His anger and guilt recently began to dissolve when he received letters from senior UN figures who acknowledged and took responsibility for failing to heed his warnings, “*I wasn't feeling the guilt of having carried the whole catastrophe… and that started to reopen the door to going back to church”* (44). Giving and receiving forgiveness facilitated healing of his horizontal and vertical relationships (45).

Forgiveness practices and interventions have been shown to be helpful in addressing mental and emotional health. Meta-analyses indicate that people receiving forgiveness interventions report more forgiveness than those with no intervention. Forgiveness has been shown to provide psychological, mental, and spiritual health benefits; afford freedom from guilt and shame; decrease anxiety, depression, and anger; and increase self-esteem, hope, and a positive disposition for oneself, others, and the Sacred (46–48). Further, a process-based intervention has been found to be more effective than a shorter cognitive decision-based model (46–49). Components of effective practices and interventions include recalling the offense, empathizing with the offender, making a choice, committing to forgive (38), taking responsibility for one's actions, and making amendments where appropriate. These components are found in MI interventions such as Spiritually-Oriented Cognitive Processing Therapy (which includes forgiveness), and the Impact of Killing program [which requires development of a personalized forgiveness plan that serves as a springboard to self-forgiveness (26)].

Forgiveness practices in the spiritual domain are rooted in S/R and cultural narratives/rituals. In addition to a reduction of negative emotions, greater peace of mind, and improved quality of life that self-forgiveness might offer, forgiveness practices found in traditional cultures and S/R traditions can also enable repentance, realignment, cleansing (29) and reconnecting with the Sacred and community. Where individuals have lost meaning and purpose, reconnection to the Sacred offers a sense of hope in the present or afterlife (50). To that end, Native American traditions offer the purification lodge, Catholicism offers confession, Judaism offers the 10 Days of Repentance, Shamanic traditions offer journeys to the spirit world, and Buddhism offers the wheel of karma (51). In the Catholic tradition's confessional model, steps to forgiveness include examination of conscience, expressing regret, naming a mistake, having a change of heart, seeking forgiveness from God, and making amends with self and others (51). Processes drawn from these traditions can support recovery, and when combined with a person-centered, biopsychosocial-spiritual approach, may help service members and veterans make sense of MI; give and receive forgiveness; and reconnect with themselves, their families, larger communities, and the Sacred (52–57).

## Repairing Relationships

Forgiveness results when relationships are made right (4, 15, 34, 35, 58–61). In the following paragraphs, we discuss three relational aspects of forgiveness: of self, to and from the other, and with the Sacred.

### Forgiveness of Self

Self-forgiveness concerns how one views oneself and aims to free the self from guilt or shame by accepting responsibility for having violated socio-cultural and S/R values and beliefs. Self-forgiveness enhances wellbeing by promoting relational repair and replacing negative condemning emotions with positive, affirming ones (62). Within the military environment, self-forgiveness can enable service members to thrive despite encountering ethical challenges (34, 63). Self-forgiveness has also been identified as a potentially important component of MI healing, with Griffin et al. theorizing that self-forgiveness may “provide a framework by which to satisfy fundamental needs for belonging and esteem that moral pain often obstructs” [(64), p. 78].

### Forgiveness to and From the Other

Interpersonally, forgiveness aims to mend relationships. Central factors of forgiveness are cultivating an empathic perspective toward the offender; genuinely wishing the offender well while releasing hurt and angry emotions; and reframing the transgression through a more cognitive and less emotionally reactive interpretation (38). Further, seeking forgiveness has been positively correlated with mental health (65, 66). Those who receive forgiveness report experiencing a sense of relief, a desire not to hurt the other again, and an improved relationship with the other (67, 68).

### Forgiveness With the Sacred

Forgiveness may also involve forgiving and/or being forgiven by that which is beyond oneself (69, 70). When exposed to PMIEs, people often express anger at the Sacred who they believe has let them down or abandoned them in their time of need. For some individuals, forgiving and receiving forgiveness from the Sacred is necessary before it is possible to heal from MI. Forgiveness by the Sacred is associated with increased self-forgiveness (67), suggesting that when a person feels forgiven, they are more able to extend compassion to themselves and others (71).

## Case Illustrations

The impact of forgiveness is best exemplified using case illustrations. These demonstrate the effect of forgiveness as it applies individually and collectively when facilitated jointly by Mental Health Professionals and S/R Leaders. In the following paragraphs, we describe two cases that offer a window into ways in which forgiveness can occur. Each case describes forgiveness practices and interventions used to facilitate a process of recovery and relational repair. While our case examples are drawn from military service members and their deployment experiences, the application of forgiveness as an intervention can extend more broadly to all affected by MI.

### Case 1

After serving 16 years, completing a final tour in Afghanistan, and being involuntarily released, a service member began experiencing night terrors, insomnia, depression, and anxiety. Relentless images associated with having taken the life of another person tormented him. At the urging of his family, he reluctantly entered therapy. During a course of cognitive processing therapy, it became clear that the primary trauma event involved the act of killing. Identifying MI and his strong feelings of anger and unforgiveness, his therapist encouraged him to meet with a S/R Leader as a complement to his therapy.

#### Forgiveness Practices

While meeting with a S/R Leader and engaging in an intensive narration of the PMIE, he disclosed that he resented his Chain-of-Command, was unable to forgive God for letting the event happen and hated himself. He expressed that he was repulsed by what he had done and struggled to reconcile his actions with who he was and his beliefs and values. His existential pain was palpable, as were his feelings of unworthiness to be in relationships with his wife, children, others, and God. He indicated that he had considered ending his life to stop the suffering. Honest and non-judgemental discourse with a S/R Leader enabled him to gradually forgive his Chain-of-Command and himself and ask for and offer forgiveness to God. He eventually engaged in a practice of reconciliation that aligned with his S/R beliefs and practices.

#### Repairing Relationship

Forgiveness allowed him to face the PMIE, reflect on its impact, extend and receive forgiveness, and find resolution and closure. This increased his ability to engage in and further benefit from mental health interventions with his therapist.

### Case 2

Veterans from a military unit, embarking on a “Return with a Mission” trip, journeyed to memorable places of their deployment. At one point, they began making their way to a location high on a steep mountain trail where one of their comrades had tragically died 25 years earlier. Arriving at the site, they affixed a commemorative plaque to a tree inscribed with their colleague's name, rank and the date of her passing. Each silently reflected on the mission, their colleague, the role they played in her life and death, as well as the moments that transpired that fateful day.

#### Forgiveness Practice

The veterans, with a MHP and chaplain among them, gathered together in a circle and engaged in a ritual at the place of her passing. Emotions ran high and tears were shed. Each person was invited to light a candle at the base of the tree, and silently contemplate the following sentiments: “I remember you and my lack of doing something that could have protected you. I ask your forgiveness and forgive myself and others for my/our omission. I release you and accept your forgiveness. I choose now to move from darkness to light.” Each then resumed their place in the circle. Testimonials were read and a prayer was offered by the group.

#### Repairing Relationship

The veterans were able to release the guilt, unforgiveness, and shame they had carried, and experienced mending of relationships with their colleague, self, others and the Sacred—re-uniting, re-membering, and becoming “one” once again. Engaging in this journey enabled the veterans to make meaning of the event, reconcile and heal in a way that they had not been able to experience before, and pursue further growth and therapeutic opportunities.

## Discussion

MI may necessitate that Mental Health Professionals adopt a different approach to trauma than is commonly used with PTSD (72). This article examined forgiveness and forgiveness practices for service members and veterans struggling with MI. By way of two vignettes, it showcased how forgiveness practices can facilitate restoration of one's sense of self, relationships with others, and for some, the Sacred. We questioned whether conventional models of evidence-based interventions for MI are lacking reference to forgiveness or forgiveness practices and may benefit from integrating these into clinical care (17, 59, 72).

Forgiveness can help individuals recognize the weight of MI and their (in)actions, release negative emotions, and mend relationships. As a discourse, however, forgiveness is commonly reserved for S/R Leaders and is not well incorporated into mental health contexts. Forgiveness practices long-employed by S/R traditions, or those that draw on S/R principles, may yield a more holistic approach to MI service-provision. Such practices may enable service members and veterans to face shame and guilt associated with actions or inactions, let go of negative emotions, and mend crucial relationships (62, 63, 71, 73, 74). Integrating forgiveness practices may facilitate healing of MI and associated conditions such as PTSD, anxiety, and depression. When incorporating forgiveness, collaboration between S/R Leaders and Mental Health Professionals would be valuable (18, 24, 53).

While some clinicians associate PTSD and MI symptoms with maladaptive cognitions (75), there is a paucity of research on cognitions associated with MI. MI may in fact signal that something one has experienced is fundamentally “wrong.” Therefore, rather than reflecting a maladaptive cognition, MI may critically reflect adaptive cognitions, with the resulting struggle arising when things are not “as they ought to be” (76). As a result, recovery can require an alternative approach to evidence-based trauma therapies.

As the literature suggests, spiritual strength programs that incorporate forgiveness concepts and practices may facilitate reconciliation and healing (77). Several examples of programs used in clinical treatment are of note. The Forgiveness Interview Protocol (FIP) is a narrative therapy writing process that utilizes distinct theoretical and clinical disciplines for mental health counseling and S/R care (78). Acceptance and Forgiveness Therapy (AFT) has recently been introduced by Pernicano et al. (79). As a psychospiritual group intervention, AFT experientially guides veterans with MI from a trauma-focused to restorative view of self. S/R Leaders and Mental Health Professionals jointly deliver psychoeducation, facilitate therapeutic interaction, and encourage home practice. The curriculum includes evidence-driven psychological interventions, spiritually-oriented practices, metaphors, stories, and art to illustrate concepts and facilitate self-expression. Another example is Forgiveness Bibliotherapy (80). The efficacy of an 8-week Forgiveness Bibliotherapy intervention with undergraduate nursing students was tested using Enrich's *8 keys to forgiveness* (81). After reading and providing a weekly reflection on each chapter, forgiveness and forgiveness-related outcome measures pre/post and one-month follow-up showed that the experimental group had significantly greater improvements in forgiveness, anxiety, depression, and fatigue. Such promising practices merit further study and implementation.

There are also limitations and cautions to the concept of forgiveness. First, forgiveness is a process. It may take time for individuals to face events at the root of unforgiveness and acknowledge actions, inactions and harms done to themselves or others. The timing of and pace at which forgiveness occurs is unique to each person, with people needing to be ready to forgive and choosing to do so. Further, for those who have experienced abuse, forgiveness necessitates particular care and an understanding that it is not necessary to engage in a relationship with an offender to forgive them, particularly if it would put them in harm's way.

It is vital to have an understanding of forgiveness from an interdisciplinary perspective. The study of the role of forgiveness in the treatment and healing of MI and complementarity of approaches to forgiveness that can be used by Mental Health Professionals and S/R Leaders is of critical importance. With mental health practices having a different discourse than S/R approaches, ways in which S/R forgiveness practices complement evidence-based interventions may be needed. For example, Mental Health Professionals tend to speak about “treatment” and “interventions,” while S/R Leaders speak about “practices” and “healing.” Such approaches can be complementary and benefit not only service members and veterans, but all those experiencing MI as a result of exposure to PMIEs.

Further research into MI is warranted and would benefit from an interdisciplinary approach. This includes study of: (i) the relationship between MI and forgiveness, (ii) S/R-informed prevention strategies, (iii) S/R components of forgiveness, (iv) types of modalities most conducive to forgiveness, and (v) the importance of healing relationships through forgiveness. While self-forgiveness as a concept is increasingly recognized, greater consideration is needed regarding additional topics such as the relationship between forgiveness and MI from victim and offender perspectives, triadic forgiveness, and relational repair with the Sacred. Moreover, to yet be distilled are the stages and elements of forgiveness (e.g., examination of conscience, penance, absolution, and recompense or restitution, and hope) specific to MI. These topics require further exploration for proper integration into practice. These considerations would deepen our understanding of forgiveness as a means of facilitating healing from MI.

## Conclusion

Various practices and interventions explore forgiveness in relation to MI. This article examined ways in which giving and receiving forgiveness can help restore one's sense of self by reconciling relationships with oneself, others, and the Sacred. We feel it is crucial to consider integrating forgiveness practices into clinical practice. Recovery from MI may require a novel and intentional interdisciplinary discourse between S/R Leaders and Mental Health Professionals. Recognition of the expertise offered by each discipline will be vital to this engagement. Advancement of the field of MI would benefit from further collaborative research by these disciplines regarding the role of forgiveness in the treatment and healing of PTSD and MI.

## Data Availability Statement

The original contributions presented in the study are included in the article. Further inquiries can be directed to the corresponding author.

## Author Contributions

SB-P, TC, LS-M, JH, and EV participated in the concept and writing of this manuscript and approved the final version of the manuscript.

## Funding

We acknowledge the Nyples-Tans PTSD Fund to Leiden University, and in-kind contributions from the Leiden University, the University of Alberta and the Canadian Armed Forces.

## Conflict of Interest

The authors declare that the research was conducted in the absence of any commercial or financial relationships that could be construed as a potential conflict of interest.

## Publisher's Note

All claims expressed in this article are solely those of the authors and do not necessarily represent those of their affiliated organizations, or those of the publisher, the editors and the reviewers. Any product that may be evaluated in this article, or claim that may be made by its manufacturer, is not guaranteed or endorsed by the publisher.
